# Attributable costs of ventilator-associated lower respiratory tract infection (LRTI) acquired on intensive care units: a retrospectively matched cohort study

**DOI:** 10.1186/2047-2994-2-13

**Published:** 2013-04-04

**Authors:** Rasmus Leistner, Linda Kankura, Andy Bloch, Dorit Sohr, Petra Gastmeier, Christine Geffers

**Affiliations:** 1 Institute of Hygiene and Environmental Medicine, National Reference Center for the Surveillance of nosocomial Infections, Charité, University Medicine Berlin, Hindenburgdamm 27, Berlin, 12203, Germany; 2 Department of Medical and Financial Controlling, Charité, University Medicine Berlin, Charitéplatz 1, Berlin, 10117, Germany

**Keywords:** Lower respiratory tract infection, Intensive care unit, Costs and length of stay

## Abstract

**Background:**

Lower respiratory tract infections (LRTI) are the most common hospital-acquired infections on ICUs. They have not only an impact on each patient’s individual health but also result in a considerable financial burden for the healthcare system. Our aim was to determine the costs and the length of stay of patients with ICU-acquired LRTI.

**Methods:**

We used a retrospectively matched cohort design, comparing patients with ICU-acquired LRTI and ICU patients without LRTI. LRTI was diagnosed using the definitions of the Centers for Disease Control and Prevention (CDC). Study period was from January to December 2010 analyzing patients from 10 different ICUs (medical, surgical, interdisciplinary). The device utilization ratio was defined as number of ventilator days divided by number of patient days and the device-associated LRTI rate was defined as number of ventilator associated LRTI divided by number of ventilator days. Patients were matched by age, sex, and prospectively obtained Simplified Acute Physiology Score II (SAPS II). The length of ICU stay of control patients needed to be at least as long as that of LRTI-patients before onset of LRTI. We used the Wilcoxon signed-rank test for continuous variables and the McNemar’s test for categorical variables.

**Results:**

The analyzed ICUs had 40,772 patient days in the study period with a median ventilation utilization ratio of 56 (IQR 42–65). The median device-associated LRTI rate was 3.35 (IQR 0.96-5.36) per 1,000 ventilation days. We analyzed 49 patients with ICU-acquired LRTI and 49 respective controls without LRTI. The median hospital costs for LRTI patients were significantly higher than for patients without LRTI (45,041 € vs. 26,467 €; p < .001). The attributable costs per LRTI patient were 17,015 € (p < .001). Patients with ICU acquired LRTI stayed longer in the hospital than patients without (36 days vs. 24 days; p = 0.011). An LRTI lead to an attributable increase in length of stay by 9 days (p = 0.011).

**Conclusions:**

ICU-acquired LRTI is associated with increased hospital costs and prolonged hospital stay. Hospital management should therefore implement control measurements to keep the incidence of ICU-acquired LRTI as low as possible.

## Introduction

Lower respiratory tract infections (LRTI) are the most frequent infections acquired on intensive care units (ICU) [1-3]. However, the incidence among ICU patients varies among different studies and depends on the definition of LRTI and the method of surveillance [2,3]. The economic impact of hospital-acquired LRTIs on healthcare systems is meanwhile an acknowledged topic in literature [2-4]. Nevertheless, there are few clinical studies assessing the attributable costs of ICU-acquired LRTIs in a DRG-based (diagnosis related groups) healthcare system [5-8]. Those studies analyzed only data from the USA. Resource utilization in the European and American healthcare systems are only partially comparable. It is unclear how those differences influence the results of economic studies. This substantiates the need for European studies. To our knowledge, this is the first study on LRTI costs in a DRG-system from Europe.

## Methods

### Setting

This study was conducted at the Charité University Medical Center, a 3,213 bed tertiary care university hospital. Approximately 136,000 patients are admitted each year and approximately 560,000 patients are treated as outpatients. The average stay in our hospital is 6.65 days and the rate of bed utilization is 83.6%. The infection control department of the Charité performs routinely surveillance for nosocomial LRTI on 10 different ICUs (medical, surgical and interdisciplinary) using the method of the German hospital infection surveillance system for intensive care units (ITS-KISS) [9,10]. ITS-KISS uses the definitions for hospital acquired infections from the Centers for Disease Control and Prevention (CDC) [11].

### Study design and data collection

We used a retrospectively matched cohort design to study costs and outcome of patients with LRTI acquired on one of our intensive care units (ICU). The study period was from January 1st 2010 to December 31st 2010. In the following, patients who acquired an LRTI while their stay on an ICU are classified as cases and patients without LRTI are classified as controls. Cases and controls were prospectively found by trained infection control nurses performing the surveillance on our ICUs for ITS-KISS. The device utilization ratio (number of ventilator days divided by number of patient days on the analyzed ICUs) and the device-associated LRTI rate (number of ventilator associated LRTIs divided by number of ventilator days on the analyzed ICUs) were calculated. Additional information for each patient was gathered by hospital file search. For all patients included in our study the following characteristics were collected: Age, sex, the Simplified Acute Physiology Score (SAPS) at entry in the ICU, the Charlson Comorbidity Index (CCI), overall length of stay, length of stay before onset of LRTI, length of stay after onset of LRTI, length of stay on the ICU and ventilation hours. The study based on secondary clinical datasets that did not include identifiable information. Therefore, further ethics statement or informed consent was not required.

### Costs and outcome

Data on hospital costs derived from true hospital costs (hospital charges) and were provided by the financial control department of the Charité University Medical Center. The analyzed costs cover the direct costs due to treatment and diagnostics and the indirect costs due to activities without patient contact (e.g. administration, hospital maintenance). The individual case charges were estimated based on definite performances and on settlement keys (e.g. nurse working time per patient). Costs were broken down into costs for medical staff, nursing staff, assistant medical technicians, medical products and for pharmacy. Daily costs were obtained by total hospital costs divided by number of hospital days. Reimbursement per patient was calculated on the basis of the diagnosis related groups (DRG), provided by the financial control department. The attributable costs and attributable length of stay (LOS) of hospital acquired LRTI were calculated as median of the differences in total costs of cases and their respective controls. The patient case weight serves as a weighing factor to give medical cases an economic dimension to demonstrate their economic severity. The case weights are estimated on the basis of several parameters: e.g. the patients’ primary and secondary diagnoses, the therapy, age, sex, ventilation time.

### Cases and definitions

Cases were defined as patients who were 18 years or older at the time of hospital admission and who acquired a LRTI or a pneumonia on one of the analyzed ICUs. Cases of pneumonia and LRTI were subsumed as LRTI cases. Cases of LRTI were diagnosed using the CDC definitions and were considered ICU-acquired if no evidence that the infection was present or incubating at the time of admission to the ICU [11]. The used CDC definitions include clinically defined pneumonia, laboratory confirmed pneumonia and lower respiratory tract infection other than pneumonia. A laboratory confirmation is required for the latter two definitions. The included patients had to be admitted and discharged within 2010. Patients’ whose stay ended later than 2010 were excluded. Mortality was defined as hospital mortality.

### Controls and matching criteria

All patients who were discharged in 2010 and who were admitted to an ICU in 2010 were considered a potential control. Patients who were younger than 18 years at the time of admission and patients who stayed less than the minimum of 3 days on the ICU were excluded. Control patients who were clinically diagnosed with a LRTI or who showed signs of LRTI in the retrospective file search were excluded as controls. Cases and controls were matched in a ratio of 1:1, using following criteria: Age (± 5 years), sex, and SAPS (± 10 points) and the total length of ICU stay of controls needed to be at least as long as that of cases before onset of LRTI.

### Statistical analysis

For the case and control patients, we calculated the median and interquartile range (IQR) for continuous parameters and number and percentage for categorial parameters. For evaluation of the matching criteria application and to test differences between groups, we used the Wilcoxon signed-rank test for continuous variables and the McNemar’s test for binary variables. Data were analyzed using PASW Statistics 18 (SPSS Inc., 2009, Chicago, Illinois). P ≤ 0.05 was considered significant.

## Results

### Characteristics of the analyzed ICUs and patients

Searching the infection control database we found a total of 90 cases of LRTI-acquired on our analyzed ICUs. The overall number of patient days on the analyzed ICUs was 40,772 and 22,893 device days, leading to a median ventilation utilization ratio of 56 (IQR 42–65) per 1,000 device days. The median device-associated LRTI rate was 3.35 (IQR 0.96-5.36) per 1,000 ventilation days. The median LOS on the ICU before onset of LRTI was 6 days (IQR 5–10). The four most commonly found organisms in cases with LRTI were *E.coli* (19%), *S.aureus* (16%), *Klebsiella spp.* (12%) and *P.aeruginosa* (7%). The only antimicrobial resistant organism was methicillin resistant S*.aureus* (MRSA), which was found in 6 cases. We found a total of 3,896 potential controls that were matched to each single LRTI case patient as described. Forty-nine patients with LRTI and 49 patients without LRTI remained for cost calculation after the matching process. Of the 49 case patients, 37 (75%) had a pneumonia whereas 21 cases were laboratory confirmed pneumonia and 16 clinically defined pneumonia. Twelve patients (25%) were diagnosed with a LRTI. The distribution of ICU types (surgical, medical, interdisciplinary) significantly varied between cases and controls (p = 0.005) is shown together with the analyzed patient characteristics in Table 1.

**Table 1:** Descriptive characteristics of cases with ICU-acquired LRTI and control patients without LRTI

**Characteristic**	**Cases (N = 49)**	**Controls (N = 49)**	P-value*
SAPS II†	48 (36–64)	50 (38–61)	0.535
Age†	63 (53–75)	63 (54–75)	0.455
Males †	30 (62%)	30 (62%)	1.000
Time with mechanical ventilation (h)	444 (182–682)	84 (0–304)	**<.001**
Surgical ICU	37 (76%)	23 (47%)	0.005
Medical ICU	6 (12%)	16 (33%)	
Interdisciplinary ICU	6 (12%)	10 (20%)	
In-house mortality	14 (29%)	2 (4%)	0.002
Charlson comorbidity index	7 (4–8)	5 (3–8)	0.350
Myokardial infarct	3 (6%)	0 (0%)	0.250
Congestive heart failure	24 (49%)	19 (39%)	0.359
Peripheral vascular disease	11 (22%)	5 (10%)	0.109
Cerebrovascular disease	23 (47%)	16 (33%)	0.265
Dementia	2 (4%)	1 (2%)	1.000
Chronic lung disease	11 (22%)	9 (18%)	0.791
Connective tissue disease	1 (2%)	0	1.000
Peptic ulcer	0	1 (2%)	1.000
Mild liver disease	6 (12%)	6 (12%)	1.000
Diabetes without complications	12 (25%)	8 (16%)	0.388
Diabetes with complications	2 (4%)	2 (4%)	1.000
Renal disease	25 (51%)	14 (29%)	**0.027**
Tumour	5 (10%)	13 (27%)	0.057
Moderate or severe liver disease	4 (8%)	5 (10%)	1.000
Malignant tumour	2 (4%)	6 (12%)	0.219
AIDS	0	1 (2%)	1.000
Hemiplegia	16 (33%)	8 (16%)	0.096
Leukemia	1 (2%)	0	1.000
Lymphoma	0	0	1.000

SAPS II, simplified acute physiology score; AIDS, acquired immunodeficiency syndrome; LOS, length of hospital stay in days.

Values are given as number (%) or median (interquartile range).

* Wilcoxon signed-rank test for continuous variables and McNemar’s test for binary variables.

† Matching criteria. Additional matching criteria were: discharge within 2010 and a length of stay at least as that of cases before onset of LRTI.

### Costs, Length of stay and case weight

The median total hospital costs for cases was significantly higher compared to the controls 45,041 € vs. 26,467 €; p < .001). The total LOS was 36 days (24–74) for cases and 24 days (18–34) for controls (p < .001) and the length of ICU stay was 28 days (16–40) for cases and 11 days (8–18) for controls (p < .001). The attributable hospital costs for ICU-acquired LRTI per patient was 17,015 € (IQR 4,531 € - 32,231 €; p < .001) and the attributable LOS for ICU-acquired LRTI was 9 days (IQR -8 days – 24 days). The analysis of detailed costs of cases vs. controls revealed significant differences for medical staff (6,458 € vs. 2,506 €; p < .001), nursing staff (10,696 € vs. 4,650 €; p < .001), assistant medical technicians (195 € vs. 87 €; p < .001), pharmacy (1,820 € vs. 593 €; p < .001) and medical products (2,712 € vs. 1,043; p < .001). The calculation of costs are shown in Table 2. The cases weight of LRTI cases was 16 (10–24) and 8 (6–10) for controls (p < .001).

**Table 2:** Costs for cases with ICU-acquired LRTI and control patients without LRTI

**Characteristic**	**Cases (N = 49)**	**Controls (N = 49)**	P-value*
Total hospital costs (€)	45,041 (30,563-62,224)	26,467 (16,899-36,488)	<.001
Reimbursement per patient (€)	47,952 (30,688-69,841)	23,013 (15,056 - 30,688)	<.001
Total ICU costs (€)	28,911 (18,294-43,890)	11,785 (6,576 - 19,207)	<.001
Medical staff (€)	6,458 (3,256-9,638)	2,506 (1,179 - 4,593)	<.001
Nursing staff (€)	10,696 (6,659-16,734)	4,650 (2,351 - 7,634)	<.001
Assistant medical technicians (€)	195 (120–274)	87 ( 51–178)	<.001
Pharmacy (€)	1,820 (950–2,554)	593 (338–1,316)	<.001
Medical products (€)	2,712 (1,500-4,464)	1,043 (513–1,835)	<.001
Daily costs (€)	1,503 (1,095-1,809)	1,070 (833–1,441)	0.001

Values are given as median (interquartile range).

* P-value, Wilcoxon signed-rank test.

On the basis of the average € / USD exchange rate for 2010 (1.00 € = 1.39 USD) we converted the median costs into USD. The total hospital costs in USD were 62,607 USD (IQR 42,483 USD - 86,491 USD) for cases and 36,789 USD (IQR 23,490 USD - 36,489 USD) for controls. The attributable costs for an LRTI per patient was 23,651 USD (IQR 6,298 USD - 44.801 USD).

## Discussion

Our study examined costs of ICU-acquired LRTIs in a DRG-based healthcare system in an university hospital. The study population consisted of ICU patients who were admitted to an ICU that participated in the German hospital infection surveillance system. We found that the development of an LRTI resulted in a prolonged hospital stay and higher attributable hospital costs than without a LRTI. Looking at the daily costs, the numbers demonstrate that the average (true) costs per day are also significantly higher in cases compared to controls. These numbers show that a prolonged LOS (even probably the most dominant factor) is not the only reason for additional hospital expenses. The significantly higher case weights for LRTI patients document the increased complexity of these cases even though underlying diseases controlled by SAPS II and Charlson Comorbidity Index were comparable between cases and controls.

Hospital-acquired LRTI is a time dependent event. The longer a patient remains in the hospital, the higher the chances for such an infection. On the other hand, costs in a DRG-system are also modulated by the length of stay. Hence we adjusted our matched cohort by time before onset of LRTI. In this way, we ensured that our data on costs and length of stay would not be overestimated [12]. A study with matched patients tries to compare patients with a similar health condition. There are many scores that can be used to adjust for comorbidities. We used the SAPS II score for matching. We obtained another comorbidity score - the Charlson Comorbidity Index (CCI) [13] - but we did not use it as a matching criterium. After the matching process, the CCI showed no significant difference and confirmed the successful adjustment for underlying diseases. Among the 90 LRTI cases, we were able to match only 54% due to strict matching criteria. However, we are convinced that we did not overmatch our cohorts since differences in risk factors - like mechanical ventilation time – are still detectable even with statistical significance. Our data therewith affirms the results of other studies that showed mechanical ventilation significantly associated with hospital-acquired LRTI [2,3,14]. The ventilation-associated LRTI rate in our cohort was lower than the reference data from Germany (635 ICUs, from 2007 to 2011) which was 4.35 (IQR 2.20-7.30) [15]. The difference could be explained by a strongly preselected patient population that is treated in our university which serves as a highly specialized tertiary care center.

In our study, the attributable costs per LRTI case were 17,015 € which equals 23,651 USD. The comparison of studies on economics of LRTIs is very difficult. Most studies use different methods and, importantly, different matching criteria. Table 3 provides an overview of relevant studies on that topic. Since different billing systems create different incentives to health care providers the billing system should be considered in medico-economic analyses. Even though the numbers on costs of LRTIs vary very much increased hospital costs have been reported for patients with LRTIs unanimously in different DRG-based studies [5-8]. A matched cohort study by Kollef et al. estimated the monetary difference of means of cases with and controls without ventilator-associated pneumonia (VAP) to be 39,828 USD [6]. However, this study retrospectively analyzed a large database, finding cases of pneumonia by DRG code. Restrepo and colleagues estimated the median costs for VAP cases vs. non-VAP controls 76,730 USD vs. 41,250 USD [7]. This would lead to an absolute difference of 35,480 USD. They discriminated cases and controls by microbiologically confirmation of VAP that were otherwise similar in respect to admission diagnosis, mechanical ventilation and other risk factors. Both methods could have led to an overestimation of costs due to a low sensitivity, detecting mostly clinically very significant cases of VAP. Warren et al. prospectively found in cohort study cases of VAP using the NNIS criteria. They calculated the attributable costs for VAP to be 48,948 USD and after adjustment for other cost influencing factors at 11,897 USD [8]. Coconour et al. showed more than 3 times higher ICU costs for cases of VAP than for controls without VAP. This lead to an absolute difference of ICU costs of 57,158 USD [5]. Even though our absolute ICU costs are significantly lower in both groups, developing a LRTI was associated with a 2.5 increase of ICU costs. A systematic review by Safdar et al. from 2005 analyzed 89 studies published after 1990, also including non-DRG based studies. Safdar computed the average additional costs for VAP between 10,019 USD and 13,647 USD [4]. They estimated attributable costs for a VAP on the basis of attributable ICU LOS of three studies that were not DRG-based. Their equation was done not by using the real hospital costs of the analyzed studies but by using cost data from the University of Wisconsin. Another important difference to those studies on costs of airway infections is that mostly VAP cases were analyzed. In our study, we also included cases of bronchitis. Since bronchitis is a less severe infection, this could have lead to overall lower costs and shorter LOS.

**Table 3:** Relevant literature to costs and length of stays (LOS) of patients with lower respiratory tract infection

**Author, Year**	**Study country**	**Study design**	**Adjusted for time – dependency bias**	**Type of infection**	**DRG-based**	**Exposed : unexposed**	**Attributable costs**	**Attributable hospital LOS**
Kollef, 2012 [6]	USA	Matched cohort, retrospectively	No	VAP	Yes	2,144 : 2,144	39,828 USD*	13 days*
Restrepo, 2010 [7]	USA	Matched cohort, retrospectively	No	VAP	Yes	30 : 90	35,480 USD*	13 days*
Cocanour, 2005 [5]	USA	Matched case control, prospectively	No	VAP	Yes	70 : 70	57,158 USD*†	14 days*†
Warren, 2003 [8]	USA	Cohort study, prospectively	No	VAP	Yes	127 : 629	11,897 USD	21 days*
This study, 2012	Germany	Matched cohort, prospectively	Yes	LRTI	Yes	49 : 49	23,651 USD	9 days

* absolute difference of mean or median of exposed and unexposed patients.

† only ICU. LRTI, lower respiratory tract infection. VAP, ventilator-associated pneumonia.

In our study, an episode of LRTI resulted in an attributable stay of 9 days in the hospital. Even though we did not detect a difference in underlying morbidity LRTI was also associated with a significantly increased mortality. However, our study confirms the results of many other studies that showed that the development of a LRTI leads to a prolonged hospital stay [5,16-19]. Those studies report an extra hospital stay between 2 and 13 days depending on the study design, the method of calculation and whether the study institutions used the DRG-system. Nosocomial LRTIs are time-dependent events. Hence studies examining LOS need to adjust for this bias [12]. DRG based studies that adjusted for time-dependency reported on median additional hospital stays of 9 to 13 days [6,7,17] due to VAP.

### Limitations

There are some limitations to our analysis. Even though we found 90 LRTI patients, we were able to match and analyze only 54% of the cases with non-exposed patients. Therefore the cohort is comparable small. Second, we did not assess the antimicrobial therapy of the analyzed patients. Therefore we could not assess the effect of an adequate and timely antimicrobial therapy on the outcome of our 49 LRTI-patients. Third, we assessed only patients from the Charité University Hospital Berlin. Therefore our results might be representative only for our institution.

## Conclusion

The attributable hospital stay for an LRTI was 9 days (p = 0.011) resulting in attributable costs per LRTI of 17,015 € (p < 0.001). Considering the economic impact and the impact on the health-care system, we strongly recommend the introduction of appropriate measurements to prevent the development of hospital acquired LRTI.

## Competing interests

The authors declare that they have no competing interests.

## Authors’ contribution

RL participated in the study design, coordination and helped to draft the manuscript. LK collected and validated the data and helped to draft the manuscript. AB participated in the study design and helped to draft the manuscript. DS participated in the study design and performed the statistical analysis. PG conceived of the study, participated in its design and coordination and helped to draft the manuscript. CG conceived of the study, and participated in its design and coordination and helped to draft the manuscript. All authors read and approved the final manuscript.
